# Complications of Mini-Percutaneous Nephrolithotomy for Staghorn Stones

**DOI:** 10.7759/cureus.94431

**Published:** 2025-10-13

**Authors:** Zafar Ahmad Khan, Qudrat Ullah

**Affiliations:** 1 Urology, Bacha Khan Medical College, Mardan Medical Complex, Mardan, PAK; 2 Urology, Institute of Kidney Diseases, Peshawar, PAK

**Keywords:** complications, mini-percutaneous nephrolithotomy, staghorn calculi, stone-free rate, urosepsis

## Abstract

Background and objective

Staghorn calculi are big, intricate kidney stones that reside in the calyceal and renal pelvis. They present serious surgical difficulties and a high risk of complications. A less invasive option to standard percutaneous nephrolithotomy (PCNL), mini-percutaneous nephrolithotomy (mini-PCNL) uses smaller tract diameters to minimize bleeding, postoperative pain, and hospitalization while preserving similar stone-free rates (SFR). However, there is still little proof of its effectiveness and safety for staghorn stones, especially in local contexts. This study aimed to assess clinical results and SFR of mini-PCNL, as well as intraoperative and postoperative complications in patients with staghorn calculi.

Methods

We conducted a prospective observational study from January 2024 to March 2025 at Mardan Medical Complex and the Institute of Kidney Diseases in Peshawar, Pakistan. Mini-PCNL was performed on 120 patients aged 15 to 70 years with complete or partial staghorn calculi. Non-contrast CT scans were used as part of the preoperative evaluation to determine renal architecture and stone burden. The Clavien-Dindo system was used to categorize complications, and SPSS Statistics version 26.0 (IBM Corp., Armonk, NY) was used to analyze the data.

Results

The mean age of the 120 patients was 42.3 ± 14.7 years, with 68 (56.7%) male and 52 (43.3%) female. Complete staghorn calculi were seen in 72 (60%) of patients. Intraoperative complications occurred in 22 (18.3%). The most common complications were hemorrhage in 10 patients (8.3%) and collecting system injury in five patients (4.2%). Postoperative complications included fever/infection (n=20, 16.7%), urosepsis (n=5, 4.2%), delayed bleeding (n=3, 2.5%), and mortality (n=1, 0.8%). The SFR was 101 (84.2%), and the average length of hospital stay was 3.8 ± 1.2 days. Secondary operations were required in 12 (10%) cases.

Conclusions

Mini-PCNL is a safe and effective approach for managing staghorn calculi, achieving high SFR with acceptable complication profiles. Optimized perioperative care and infection control remain critical for favorable outcomes.

## Introduction

Staghorn calculi present in two main forms, partial and complete. Partial staghorn stones are confined to the renal pelvis and at least two calyces, whereas complete staghorn calculi occupy more than 80% of the collecting system, extending into the renal pelvis and calyceal branches [1]. Percutaneous nephrolithotomy (PCNL) remains the recommended treatment for large-volume and staghorn calculi according to contemporary guidelines [2,3]. Despite advances in endourological techniques and instrumentation, the frequency of PCNL performed for staghorn calculi has remained relatively stable over time, as reported in contemporary multicenter and registry-based studies [1,2]. Compared with non-staghorn stones, these calculi are associated with higher perioperative complication rates and remain a considerable challenge for endourologists [4].

Evidence from the Clinical Research Office of the Endourological Society (CROES) demonstrated that patients undergoing PCNL for staghorn calculi achieved a stone-free rate (SFR) of 3036 (56.9%) compared with 4401 (82.5%) in patients with non-staghorn stones [5]. In addition to technical challenges, insufficient communication between patients and urologists regarding expected outcomes and complications may further complicate management and increase the likelihood of medico-legal disputes [6]. Tools such as structured scoring systems, audiovisual aids, and informational booklets can improve communication, support shared decision-making, and help establish realistic patient expectations [7-12].

A comprehensive preoperative evaluation that considers patient comorbidities, stone characteristics, and renal anatomy is critical for predicting and managing potential complications of PCNL in staghorn cases. Clinical factors such as obesity, higher BMI, and frailty index, along with stone-related parameters including stone size, density, and complexity, can help predict postoperative outcomes and the likelihood of complications. Non-contrast CT remains the gold standard for assessing stone burden and anatomical parameters [13]. CT imaging provides essential details, including skin-to-stone distance, relationship of calculi to adjacent viscera [14], and infundibular neck width, which determines the feasibility of percutaneous access, instrument maneuverability, and the overall likelihood of achieving complete stone clearance [15-17]. In addition, the angles between stone branches and puncture entry sites, overall stone volume, and stone density are critical predictors of procedural complexity and outcomes [9,15,16]. To optimize anatomical assessment, some authors recommend performing CT imaging with the patient in the anticipated surgical position, either prone or supine, thereby allowing more accurate planning of access routes [18].

Mini-percutaneous nephrolithotomy (mini-PCNL), which uses smaller tract sizes, has been developed as a modification to reduce morbidity while maintaining high stone clearance. Nevertheless, both intraoperative and postoperative complications remain clinically significant in staghorn populations. Hemorrhage, although uncommon, may occur due to vascular injury during tract dilation, sometimes requiring embolization [19]. Other intraoperative risks include mild fever, puncture site oozing, abdominal ileus [19], transfusion requirements [20], collecting system injuries [21], and, in rare cases, severe complications such as pelvic avulsion or extravasation necessitating surgical intervention [20]. Studies have shown high irrigation volumes precipitating hypothermia in 56.2% and cardiovascular instability in 57.1% of patients, while increased intrarenal pelvic pressures during mini-PCNL elevate the risk of systemic absorption, postoperative fever, and inflammatory response [21-23]. Intraoperative bleeding can further compromise visualization and prolong operative time [24,25].

Postoperative complications are likewise important to consider. Urinary tract infections occur in up to 37% of PCNL cases and may progress to urosepsis in 0.3-7.6%, with reported mortality rates as high as 66-80% among patients who develop severe sepsis [26,27]. Early bleeding within 24 hours or delayed bleeding (n=17, 1.2%) of cases may arise from vascular injuries, whereas persistent nephrocutaneous leakage beyond 24 hours is seen in 21-65 (1.5-4.6%) cases [27]. Infundibular stenosis, though rare (n=28, 2%) [28], may occur due to inflammatory scarring, particularly when large nephroscopes are used relative to calyceal diameter. Treatment options include endoscopic techniques such as cold-knife incision, balloon dilation, or laser ablation, with reported success rates of 844-1125 (60-80%) [27]. Finally, mortality after PCNL remains low at approximately three (0.2%) in large cohorts, with sepsis representing the most frequent cause of death [27].

## Materials and methods

This prospective observational study was carried out at the Institute of Kidney Diseases and Mardan Medical Complex in Peshawar, Pakistan, between January 2024 and March 2025. A total of 120 patients diagnosed with staghorn calculi were included, with Mini-PCNL performed in patients having a stone size of more than 1.5 cm in the lower pole and more than 2 cm in the pelvis and mid or upper pole. Participants, comprising both male and female patients, ranged in age from 15 to 70 years. Under general or regional anesthesia, all patients underwent mini-PCNL using standardized surgical procedures. Preoperative evaluation included complete medical history, physical examination, standard laboratory investigations, and radiographic imaging such as non-contrast CT (NCCT) to assess renal anatomy, stone size, and burden.

Intraoperative variables such as operative time, number of tracts used, and complications were recorded. Postoperative monitoring was performed to identify early and late complications, which were classified using the Clavien-Dindo system. Patients with complete or partial staghorn calculi were included if considered suitable for mini-PCNL, defined by a stone size of more than 1.5 cm in the lower pole and more than 2 cm in the pelvis, mid, or upper pole, with favorable renal anatomy and no active urinary tract infection at the time of surgery, while those with significant comorbidities contraindicating surgery, uncontrolled urinary tract infections, or uncorrected coagulopathies were excluded.

Ethical approval for this study was obtained from the Institutional Research and Ethical Committee of the Institute of Kidney Diseases, Hayatabad Medical Complex, Peshawar (Ref No: 445/Chairmen/R&E/Committee/IKD, dated 1st December 2023). Written informed consent was obtained from all patients before their inclusion in the study, in accordance with the Declaration of Helsinki.

Data were collected using a structured proforma and analyzed with SPSS Statistics version 26.0 (IBM Corp., Armonk, NY). Demographic information, stone characteristics, and complication rates were summarized using descriptive statistics. Continuous variables were expressed as mean ± standard deviation (SD), and categorical variables were presented as frequencies and percentages.

## Results

The study included 120 patients with a mean age of 42.3 ± 14.7 years; 68 were male (56.7%), and 52 were female (43.3%). A substantial number of participants (n=42, 35.0%) were between the ages of 30 and 44, followed by those between the ages of 45 and 59 (n=33, 27.5%), 15 and 29 (n=30, 25.0%), and 60 and 70 (n=15, 12.5%) (Table 1). In terms of stone features, 48 patients (40.0%) had partial staghorn calculi, whereas 72 patients (60.0%) had entire staghorn calculi. Only five (4.1%) cases had bilateral involvement, with the right kidney (n=65, 54.2%) having the stones more frequently than the left (n=50, 41.7%) (Table 2).

**Table 1:** Demographic characteristics of patients (n=120)

Variable	Categories	Frequency (n)	Percentage (%)
Age group, years	15–29	30	25.0
	30–44	42	35.0
	45–59	33	27.5
	60–70	15	12.5
Gender	Male	68	56.7
	Female	52	43.3

**Table 2:** Stone characteristics

Variable	Categories	Frequency (n)	Percentage (%)
Stone type	Complete staghorn	72	60.0
	Partial staghorn	48	40.0
Stone laterality	Right	65	54.2
	Left	50	41.7
	Bilateral	5	4.1

According to intraoperative findings, each patient had an average of 1.3 ± 0.5 tracts and an average operating length of 95 ± 22 minutes. Of note, 22 patients (18.3%) experienced intraoperative complications, with bleeding being the most common (n=10, 8.3%), followed by thromboembolic events (n=2, 1.7%), collecting system injury (n=5, 4.2%), neighboring organ injury (n=3, 2.5%), and pneumothorax (n=2, 1.7%) (Table 3).

**Table 3:** Intraoperative findings and complications SD: standard deviation

Parameter	Values
Operative time, minutes, mean ± SD	95 ± 22
Tracts per patient, mean ± SD	1.3 ± 0.5
Intraoperative complications, n (%)	22 (18.3%)
Bleeding, n (%)	10 (8.3%)
Collecting system injury, n (%)	5 (4.2%)
Adjacent organ injury, n (%)	3 (2.5%)
Pulmonary complications, n (%)	2 (1.7%)
Thromboembolic events, n (%)	2 (1.7%)

The Clavien-Dindo system was used to categorize postoperative complications. Twenty (16.7%) patients experienced fever or mild infections (Grades I-II), while five (4.2%) patients experienced urosepsis necessitating more advanced care (Grades III-IV). Infundibular stenosis was observed in two (1.7%) cases, diagnosed during postoperative follow-up using imaging modalities such as contrast-enhanced CT urography or retrograde pyelography, which demonstrated narrowing of the infundibulum with associated calyceal dilatation. Persistent nephrocutaneous leakage occurred in four (3.3%) cases, and delayed bleeding was seen in three (2.5%) cases. One patient (0.8%) died as a result of severe septic complications (Table 4).

**Table 4:** Postoperative complications (Clavien–Dindo classification)

Complication	Frequency (n)	Percentage (%)
Fever/infection (Grade I–II)	20	16.7
Urosepsis (Grade III–IV)	5	4.2
Delayed bleeding	3	2.5
Persistent urinary leakage	4	3.3
Infundibular stenosis	2	1.7
Mortality (Grade V)	1	0.8

The average hospital stay following surgery was 3.8 ± 1.2 days. Twelve patients (10.0%) required further procedures, such as extracorporeal shock wave lithotripsy (ESWL) or repeat PCNL, for residual fragments. The mean stone size among these patients was 3.4 ± 0.6 cm, which was relatively larger than the average size in the overall study cohort. This suggests that applying stricter stone size selection criteria for mini-PCNL might help minimize the need for secondary interventions. The overall SFR following mini-PCNL was 84.2% (Table 5).

**Table 5:** Postoperative outcomes SD: standard deviation

Outcome	Value
Hospital stay, days, mean ± SD	3.8 ± 1.2
Stone-free rate, %	84.2%
Secondary procedure required, n (%)	12 (10.0%)

## Discussion

In this double-centered cohort of 120 patients undergoing mini-PCNL for staghorn calculi, we found a 12 (10%) secondary-procedure rate, a mean hospital stay of 3.8 ± 1.2 days, and an 84.2% SFR. These results are mostly in line with prior studies [28] showing that mini-PCNL achieves SFRs comparable to traditional PCNL while providing a good perioperative morbidity profile. Our safety and efficacy results are consistent with a 2025 systematic review that only looked at staghorn calculi and found no significant difference in SFR between mini- and regular PCNL, but preferred mini-PCNL for hemoglobin drop and transfusion rates [28].

Historically, the CROES global PCNL study reported substantially lower SFRs for staghorn stones 3036 (56.9%) compared with non-staghorn stones 4401 (82.5%), highlighting the intrinsic complexity of staghorn disease irrespective of approach. Our 84.2% SFR compares favorably to that benchmark and likely reflects refinements in preoperative CT planning, access strategy, and lithotripsy technologies accrued since the CROES era [29]. In addition, multiple meta-analyses comparing mini- versus standard PCNL across mixed stone burdens show similar SFRs but less bleeding, transfusion, and postoperative leakage with mini-PCNL advantages attributable to smaller tract diameters that reduce parenchymal trauma without compromising clearance when multi-quadrant access and adjunctive fragmentation are used judiciously [30-33].

Our mean hospital stay (3.8 days) is in line with pooled analyses demonstrating shorter admissions after mini-PCNL than standard PCNL, an effect generally ascribed to reduced postoperative pain, lower drain requirements, and fewer bleeding-related interventions. Several comparative series and meta-analyses consistently document earlier mobilization and discharge with mini-PCNL, reinforcing the health-system value of miniaturized tracts in appropriately selected patients [30,31,33].

Complication patterns in our cohort mirror well-described PCNL risks but remained within acceptable ranges for staghorn disease. We observed fever/minor infection in 20 (16.7%) and urosepsis in five (4.2%) patients. Large reviews place postoperative fever after PCNL in the 32-48. (20-30%) range and sepsis in roughly 0-12 (0.3-7.6%), with higher risk in infection-related stones, larger burdens, and multiple tracts-features commonly present in staghorn cohorts. Our figures sit within these intervals and underscore the need for stringent antimicrobial stewardship and intrarenal pressure control during mini-PCNL, where smaller sheaths can elevate renal pelvic pressures if outflow is inadequate [34-36]. Recent prognostic work has also reported sepsis/SIRS rates of eight (5%) in real-world series and introduced nomograms to individualize risk, tools that may guide perioperative antibiotic and access strategies in future practice [37].

Bleeding outcomes were favorable in our study. The blood transfusion rate and decline in hemoglobin were low, which is consistent with findings from comparative studies demonstrating reduced hemoglobin drop and transfusion requirements with mini-PCNL compared to standard PCNL. Mechanistically, optimizing dusting and suction-assisted evacuation while maintaining a smaller tract size (≤20 F) minimizes parenchymal trauma and shortens tamponade length, thereby reducing hemorrhagic risk without increasing residual fragments [30,31,33]. In contrast, classic PCNL studies have reported overall complication rates of up to 133 cases (83%) when minor events are included, transfusion rates ranging from 18 to 29 cases (11-18%), and rare but serious vascular or thoracoabdominal injuries. Our findings indicate that mini-PCNL may reduce the bleeding risk associated with standard PCNL, even in patients with complex staghorn calculi.

A secondary-procedure rate of 10% in our series compares favorably with multicenter experiences reporting 13-24 cases (8-15%) of ancillary interventions after mini-PCNL for complex stones [30]. The need for staged or adjunctive therapy (e.g., ESWL, second-look PCNL, or flexible ureteroscopy) reflects the anatomic reach challenges of staghorn branches and the trade-offs inherent to smaller working channels. Strategy adaptations such as planned multi-tract mini-PCNL in a single session for high-volume branching stones have been shown to preserve SFR without markedly increasing morbidity, and may further reduce residuals in selected cases [30].

Mortality in our cohort was low (n=1, 0.8%) and driven by severe sepsis, which remains the principal cause of death after PCNL in modern series. Large reviews continue to list septic events and uncontrolled hemorrhage as the chief life-threatening complications, with overall mortality typically below two (1%). Our finding is therefore consistent with the broader safety profile of PCNL while reiterating that infection-mitigation measures (preoperative urine sterilization, selective nephrostomy drainage, low-pressure irrigation, and prompt hemodynamic escalation when indicated) are pivotal in staghorn disease [34,36].

Mini-PCNL represents a successful first-line treatment option for selected staghorn calculi, particularly in cases with a moderate stone burden that can be accessed through downsized tracts and managed under controlled intrarenal pressure. The 3.8-day hospital stay and low transfusion rates in our series are consistent with the meta-analytic benefits of tract downsizing, and our SFR of around 85% aligns with the upper range reported for staghorn-directed mini-PCNL. Infectious complications remain the predominant concern; our cohort’s sepsis rates underscore the challenge of treating infection-related staghorn stones, where significant biofilm and endotoxin loads may exist. Current evidence supports several future directions, including intraoperative pressure monitoring to prevent spikes during fragmentation, risk-stratified antibiotic strategies based on predictive algorithms, and broader use of suction-enabled sheaths to maintain low intrapelvic pressures [30,31].

Finally, comparison with the CROES global experience underscores how technique evolution can narrow the historical performance gap in staghorn disease. While staghorn morphology remains a key negative predictor of SFR and a driver of complications, modern mini-PCNL with selective multi-tract strategies and careful imaging-based access planning appears capable of achieving high clearance with acceptable morbidity in this demanding population. Prospective multicenter studies focused exclusively on staghorn anatomy, and incorporating standardized pressure metrics and sepsis endpoints, would help validate these observations and refine selection algorithms for mini- versus standard PCNL in day-to-day practice [29].

The key comparative takeaways are as follows: (i) SFR: our 84.2% aligns with staghorn-specific mini-PCNL meta-analysis and exceeds legacy staghorn benchmarks; (ii) safety: while this study did not directly compare mini-PCNL with standard PCNL, existing evidence indicates that mini-PCNL generally reduces bleeding and transfusion requirements; (iii) hospital stay: shorter durations have been consistently reported in previous studies; (iv) infection: it remains the primary modifiable risk, emphasizing the need for strict pressure and antimicrobial control [28,31].

Limitations

This study has certain limitations that should be acknowledged. First, it was conducted at two centers within a single region, which may limit the generalizability of the findings to broader populations. Second, the sample size, although adequate for descriptive analysis, may not capture all potential variations in outcomes, particularly rare complications. Third, the study was observational and lacked a direct comparative arm against standard PCNL, which restricts the ability to draw definitive conclusions about relative efficacy. Additionally, long-term follow-up for stone recurrence and renal function preservation was not performed. Finally, intraoperative and postoperative care practices may have varied slightly between surgeons, potentially introducing bias. Future large-scale, multicenter studies with standardized protocols and extended follow-up are needed to validate these results.

## Conclusions

Mini-PCNL showed a good safety profile and a high SFR (84.2%) in this prospective double-centered study of 120 patients at the Institute of Kidney Diseases, Peshawar, and Mardan Medical Complex. Despite the inherent complexity of staghorn stones, the rates of complications, such as intraoperative bleeding (n=10, 8.3%), postoperative infection (n=20, 16.7%), and urosepsis (n=5, 4.2%), stayed below acceptable bounds and were in line with findings from other countries. Sepsis was the leading cause of death, which occurred in only one (0.8%) patient. These findings lend credence to mini-PCNL as a less morbid and more successful option than regular PCNL, especially when paired with infection control protocols, regulated intrarenal pressures, and careful preoperative imaging. To confirm these results, researchers should improve patient selection and evaluate long-term outcomes such as recurrence rates and renal function preservation. More multicenter studies with extended follow-up are advised.
